# Evaluation of the Protection Ability of a Magnesium Hydroxide Coating against the Bio-Corrosion of Concrete Sewer Pipes, by Using Short and Long Duration Accelerated Acid Spraying Tests

**DOI:** 10.3390/ma14174897

**Published:** 2021-08-28

**Authors:** Domna Merachtsaki, Eirini-Chrysanthi Tsardaka, Eleftherios Anastasiou, Anastasios Zouboulis

**Affiliations:** 1 Laboratory of Chemical and Environmental Technology, Department of Chemistry, Aristotle University of Thessaloniki, 54124 Thessaloniki, Greece; meradomn@chem.auth.gr; 2 Laboratory of Building Materials, Department of Civil Engineering, Aristotle University of Thessaloniki, 54124 Thessaloniki, Greece; extsardaka@gmail.com (E.-C.T.); elan@civil.auth.gr (E.A.)

**Keywords:** concrete bio-corrosion, sulfuric acid corrosion control, magnesium hydroxide coating, sewerage pipe systems, acid spraying test

## Abstract

The Microbiologically Induced Corrosion (MIC) of concrete sewer pipes is a commonly known problem that can lead to the destruction of the system, creating multiple public health issues and the need for costly repair investments. The present study focuses on the development of a magnesium hydroxide coating, with optimized properties to protect concrete against MIC. The anti-corrosion properties of the respective coating were evaluated by using short and long duration accelerated sulfuric acid spraying tests. The coating presented satisfying adhesion ability, based on pull-off and Scanning Electron Microscopy (SEM) analysis measurements. The surface pH of the coated concrete was maintained at the alkaline region (i.e., >8.0) throughout the duration of all acid spraying tests. The consumption of the coating, due to the reaction (neutralization) with sulfuric acid, was confirmed by the respective mass and thickness measurements. The protection ability of this coating was also evaluated by recording the formation of gypsum (i.e., the main corrosion product of concrete) during the performed tests, by X-ray Diffraction (XRD) analysis and by the Attenuated Total Reflectance (ATR) measurements. Finally, a long duration acid spraying test was additionally used to evaluate the behavior of the coating, simulating better the conditions existing in a real sewer pipe, and the obtained results showed that this coating is capable of offering prolonged protection to the concrete substrate.

## 1. Introduction

The good operation and efficient protection of sewerage pipeline systems, especially against MIC, is majorly important for urbanized societies. MIC is a specific type of corrosion mechanism that can take place usually in the larger diameter sewer pipes, constructed mainly from concrete, expected to highly affect the concrete and reduce the lifetime of the structure. This phenomenon has been widely studied during the last 100 years [1,2,3,4,5] and the scientific community has been focusing on the application of different mitigation techniques in order to block the collapse of the sewerage system and to prevent the costly repair/replacement of affected sewer pipes [6].

MIC is a complex corrosion mechanism, depending on the multiple chemical and biochemical processes which can take place in the sewer pipeline. Sulfates, along with other substances carried within wastewater, initiate the growth of microbial communities, which in turn cause the reduction of sulfates to hydrogen sulfide. The formed hydrogen sulfide (gas) is released to the liquid phase (wastewater) and finally, it is emitted in the upper (air) part of the (usually semi-filled) pipe. Then, the gas hydrogen sulfide can be dissolved in the humidity film located on the upper region of the pipe wall (usually denoted as “crown”) [7,8]. As a result, the pH of the concrete’s surface becomes less alkaline, and at the critical pH value of 9, Neutrophilic Sulfur Oxidizing Bacteria (NSOB) can be developed, using the hydrogen sulfide to produce biogenic sulfuric acid [9,10]. As a result the surface pH of concrete further lowers towards acidic values, until the development of Acidophilic Sulfur Oxidizing Bacteria (ASOB) at pH value 4 [10,11]. The development of these microorganisms onto the inner concrete pipe surface can lead to the production of additional biogenic sulfuric acid. The extended formation of biogenic sulfuric acid is crucial for the overall structure’s preservation and stability, because the alkaline components of concrete are reacting with acid and being consumed, forming corrosion by-products, such as gypsum and ettringite [8,10]. Finally, the concrete degrades further and the construction collapses, leading (among others) to major odor and pollution problems (e.g., leaking wastewater, polluting soil, etc.) [12,13].

The prevention methods developed so far may include the application of linings and coatings [14,15,16], located onto the inner side of sewer pipes, as well as the use of new/improved pipe materials with intrinsic anti-corrosion properties [17,18], or the appropriate chemical dosing of specific agents directly in the wastewater stream [19,20]. According to the relevant literature linings and coatings present some advantages in respect to other mitigation technologies. More specifically, coatings can create a barrier between the concrete sewer and the corrosive environment, hence offering a good protection. Additionally, the application of coatings is relatively cheaper, when compared to the replacement of destructed sewer pipes with new pipes. Finally, the environmental concerns that the alternative protection method of chemical dosing with different reagents may lead to, seem to be avoided when lining and coatings are used [21,22].

Certain coatings and linings have been already widely used for the protection of concrete [15,16,23] or steel structures (such as manganese hydrate coatings) [24]. These methods can protect the concrete surface either by blocking the interaction of substrate with the corrosive environment, or by reacting (e.g., neutralization) with the corrosive substances. Multiple protective coatings have been used for protection against MIC, according to the relevant literature, such as polymer-based (epoxy, polyurethanes), inorganic coatings (magnesium hydroxide coatings, alkali-activated materials), or cementitious coatings modified with polymers [25]. All these coatings may present specific advantages and disadvantages regarding their performance, by offering different kinds/types of protection [10]. Polymeric-based coatings isolate the concrete from the aggressive environment, without, however, reacting to or neutralizing the biogenic produced acid. Magnesium hydroxide coatings can neutralize the biogenic sulfuric acid, and can diminish it in the sewerage environment; therefore, the acid cannot initiate any secondary kind of corrosion, as compared to the polymeric-based coatings, where the acid continues to exist in the system [26,27]. The main properties of magnesium hydroxide, when applied as corrosion protection coating, are its ability to maintain for sufficient time the alkaline surface pH values of concrete substrate, hence blocking the development of sulfur oxidizing bacteria, and its ability to react (neutralize) with the produced biogenic sulfuric acid in case of microorganisms’ development.

This study is mainly focused on a specific magnesium hydroxide (MH) slurry, which after preliminary experiments was found to present the optimal expected properties, e.g., regarding the specific surface area (SSA) and the particle size distribution (PSD) [28]. The magnesium hydroxide is evaluated mainly regarding its action as protective coating on concrete substrates, under conditions that simulate those existing in the sewer pipes. The coating’s properties were additionally evaluated by examining the formation of gypsum as the main concrete corrosion by-product. The aim of this paper was to confirm that the initial physicochemical properties of used MH can affect and enhance the properties of the protective coating. The originality of this research is based on the use of a long duration acid spraying test (for four months), which is closer to the real conditions existing in a sewer pipe, especially when compared with the accelerated tests of smaller duration. Moreover, the examined concrete specimens were also replaced by proper poly(methyl methacrylate) (PMMA) substrates in order to isolate the possible side effects of concrete presence and to study the coating’s behavior under the acidic environment conditions (i.e., reacting alone with sulfuric acid).

## 2. Materials and Methods

### 2.1. Substrates

As far as concrete specimens are concerned, concrete type MC (0.45) was produced, according to the ΕΝ 1766:2017 Standard [29], conforming to the requirements of the ΕΝ 1916:2002 Standard [30], aiming the substrate to resemble the corresponding concrete materials, used for the construction of sewer pipes. The water/cement ratio was 0.45 and the proportions of the used constituents were: 410 kg/m^3^ CEM I 42.5 R, 895 kg/m^3^ crushed limestone sand (0–4 mm), 895 kg/m^3^ crushed limestone aggregates (4–8 mm), 184.5 kg/m^3^ tap water and 0.5% wt. of cement poly-carboxylate based super-plasticizer. Wooden molds with different dimensions (i.e., 200 mm × 200 mm × 20 mm and 50 mm × 50 mm × 20 mm), depending on each test specific requirements, were used for the preparation of specimens. After 24 h the concrete specimens were de-molded and cured into water for 27 days at 20 ± 2 °C.

The use of concrete specimens, as substrates for the examined protective coating, may obstruct the observation of the coating’s consumption, occurred due to the potential (neutralization) reaction of sulfuric acid with the cement (alkaline) paste of concrete. Therefore, in this study, poly (methyl methacrylate) (PMMA) plates were alternatively used as substrates, apart from the concrete substrates, in order to promote a clear observation of the coating reaction with the sulfuric acid. PMMA plates with dimensions of 50 mm × 50 mm × 5 mm were cut and used for the detailed examination of coating behavior under similar (environmental) conditions.

### 2.2. Surface Coatings

#### 2.2.1. Magnesium Hydroxide Slurry

The magnesium hydroxide slurry was provided by Grecian Magnesite S.A. It was produced in the laboratory by the controlled hydration of micro-crystalline Caustic-Calcined Magnesia (CCM), of medium purity. The MH slurry has a solids content of 57.5%, specific gravity of 1.46 g/cm^3^, a viscosity of 2500 cP (R5 spindle @100 rpm), and contains 0.4% on solids of a modified methyl cellulose as convenient adhesive material.

The main physicochemical characteristics of magnesium hydroxide solids of the slurry are provided in Table 1. The total mass loss at 1000 °C (i.e., the Loss on Ignition, LOI) corresponds to all the water and CO_2_ content of the respective powder.

This magnesium hydroxide differs mainly in the MgO content, but also in the specific surface area and in the particle size from other examined magnesium hydroxides, previously investigated [28]. The main difference regards the specific surface area, which is very low in this case (7 m^2^/g) to maintain favorable slurry characteristics, such as viscosity and workability and to allow the inclusion of cellulose. The results of the previous study [28] indicate that the bigger particle size may lead to slower interactions with the respective (acidic) environment. In that way, this magnesium hydroxide has finer particles, than other examined relevant hydroxides [28]. Additionally, the purity of raw CCM used to prepare the studied slurry, was higher, and according to previous results [28], this fact may enhance the preservation of alkaline pH values in the protected surface after the application of this coating.

#### 2.2.2. Coating Application

The thickness of relevant protective coatings, according to the literature and preliminary testing, can range from few micrometers to few millimeters, depending on the coating’s nature [23,31,32]. In this study the thickness was selected to be between 1.0–1.5 mm and was designated according to the specific amount of applied coating, i.e., 0.0018–0.0020 g/mm^2^. After the application of coating onto the substrates, the specimens were dried for 3 days under normal laboratory conditions (i.e., 21 ± 2 °C and relative humidity 60 ± 10%) before further testing.

The examined coating was also evaluated for its adhesion ability onto the concrete, by applying the pull-off bond testing method, according to the standards ΕΝ 1542:1999 [33] and ΕΝ 13578:2003 [34]. The coating was applied on concrete specimens with dimensions of 200 mm × 200 mm × 20 mm in order to perform the respective measurements. According to these standards, the pull-off equipment (digital pull-off strength tester, Matest) was used in order to record the failure load, as well as the specific type of failure.

### 2.3. Scanning Electron Microscopy Analysis

The adhesion of coating onto the concrete surfaces was also examined by Scanning Electron Microscopy (SEM) analysis, providing a closer examination of the respective concrete-coating interface and of the coating’s morphology. Moreover, specific information regarding the magnesium content, which might penetrate the concrete structure, can be obtained by using the EDS analysis. The specimens were cut vertically in order to reveal the interface between the concrete and the coating and were polished appropriately, creating a flat surface for clear observation. Micrographs were obtained by using a JSM-7610F Plus, JEOL, scanning electron microscope and the EDS analysis was performed by using the AZtec Energy Advanced X-act System (Oxford Instruments, Oxfordside, UK).

### 2.4. Accelerated Spraying Tests

According to a relevant study [28] two different accelerated sulfuric acid spraying tests were applied, so that coatings can be properly evaluated in terms of the protection they can offer to concrete surfaces in relatively short time period. The same tests and conditions were also used in this study in order to examine the selected magnesium hydroxide, presenting optimized properties. In particular, the coating was firstly examined on concrete substrates, by using a hand-held spraying device (HHD), which prayed sulfuric acid onto the coated concrete surface. Then, the coating was examined, applied onto concrete specimens in a custom-made spraying laboratory chamber, as described in [28]. In both tests, the coated specimens were placed vertically in order to simulate the real pipe walls. The laboratory chamber was constructed from poly (methyl methacrylate) and it was equipped with nebulizers and appropriate supports placed opposite them, in order to place the concrete specimens vertically. Additionally, the respective coating was evaluated in the acid spraying chamber, by applying it onto the PMMA plates, in order to evaluate the protecting properties of the coating, by eliminating the side effects of corroded concrete substrate. Finally, the spraying chamber was also used for a longer duration acid spraying test, so that the durability and effectiveness of this coating could be examined under more realistic conditions, using daily a lower acid amount.

For the application of the coating onto the concrete substrate, concrete specimens with dimensions of 50 mm × 50 mm × 20 mm were used for the acid spraying tests. Moreover, PMMA plates with the same dimensions were also comparatively used for the respective tests.

The surface pH values, the mass change and the existing mineralogical phases were recorded for all performed tests. However, the thickness change of the coating was examined only after the HHD test. The spraying tests and the measurements applied for each case are summarized in Table 2.

In all acid spraying tests a flat surface pH electrode (Extech PH100: Waterproof ExStik pH meter, Extech Instruments, Nashua, NH, USA) was used to record the surface pH values of specimens. The initial surface pH values were recorded for each specimen before the beginning of acid spraying tests. The surfaces to be measured were wetted with 1 mL of deionized water prior to the measurement.

An electronic balance Kern PCB 350–3 (350 ± 0.001 g) was used to record the mass change of specimens daily, in order to evaluate the consumption of the coating. Firstly, the initial weight of all uncoated concrete specimens was recorded, as well as their weight after the application of the slurry, in order to calculate the mass of dry coatings.

A Dino Lite Digital Microscope was used to photograph the cross-section of coated concrete specimens before and after the application of acid spraying, in order to record the thickness change of the coating. After that, the photographs were digitized and processed by using the microscope’s software and finally, the thickness was calculated according to the magnification used in each picture.

The mineralogical phases were evaluated by using the X-ray diffraction analysis (XRD), which is described in Section 2.5.

#### 2.4.1. Stoichiometry Calculations

Sulfuric acid solutions were used in all performed tests, aiming to simulate the biogenic sulfuric acid produced in a sewer pipe internal wall. The sulfuric acid spraying tests were performed to examine the total consumption of magnesium hydroxide coating at the end of respective tests, due to the neutralization reaction between them:

(1) $$\mathrm{Mg}{\left(\mathrm{OH}\right)}_{2}+H_{2}{\mathrm{SO}}_{4}\to {\mathrm{MgSO}}_{4}+2H_{2}O$$

In that way, the amount of daily sprayed sulfuric acid (according to the reaction’s stoichiometry) was calculated, based on the amount of applied coating, on the duration of each test and on the used acidic solution concentration.

The duration of accelerated acid spraying tests was selected to be 4 days, apart from the long duration test, which lasted 128 days. The used sulfuric acid solutions had concentrations of 4 M, 0.2 M and 0.1 M for the HHD spraying test, the 4-day chamber test and the 4-month chamber test, respectively. In all these experiments the same conditions were applied for the calculation of corresponding necessary sulfuric acid amount to be sprayed, as explained in more detail in the relevant literature [32].

The calculated stoichiometry (i.e., the moles of sulfuric acid) was divided by the number of spraying days and the daily spraying application stoichiometry corresponded to 25% of the overall stoichiometry, regarding the respective neutralization reaction between the coating and the sulfuric acid for the 4-day tests (i.e., the HHD spraying test and the chamber spraying tests). The daily spraying applications were different between these two tests, due to the different initial acid solutions concentrations used. Accordingly, the daily spraying application stoichiometry for the longer duration test (128 days) corresponded to the 0.78% of the overall stoichiometry of the respective reaction.

#### 2.4.2. 4-Days Accelerated Acid Spraying Test

Two tests were used in the case of 4-days accelerated acid spraying tests, i.e., the test where the hand-held device was used and the test where the custom-made poly (methyl methacrylate) chamber was used. The concentration of sulfuric acid solution in the HHD test was 4 M, in order to magnify the consumption of coating, due to the reaction with sulfuric acid, whereas the concentration of the solution in the chamber tests was 0.2 M, in order to approach the milder conditions commonly existing in a sewer pipe.

Coated concrete specimens were used in both tests, whereas the coated PMMA plates were only tested in the spraying chamber test. The coating was dried after its application for 3 days under normal laboratory conditions before the sulfuric acid spraying applications. Four coated specimens were removed daily from each acid spraying procedure and studied in detail.

#### 2.4.3. 4-Months Accelerated Acid Spraying Test

The long-term durability of the examined coating was evaluated by a 4-month accelerated acid spraying test that was held in a custom-made poly (methyl methacrylate) spraying chamber. Τhe rinsing liquids were collected separately for each specimen (chamber facility) in order to be further examined. The concentration of sprayed sulfuric acid (0.1 M) was selected in this case to be lower in respect to the 4-day tests (0.2 M), in order to simulate better the conditions existing in a sewer pipe. The longer duration test allowed the spraying of significantly lower amount of sulfuric acid daily (according to the number of daily spraying applications). In that way, the behavior and consumption of coating in time can be examined.

At 8-days intervals, four specimens were removed from the chamber and tested, regarding the surface pH values, the mass change and the mineralogical phases existing on the surface. Moreover, the rinsing liquids were selected during the same time periods, in order to determine the magnesium content that was rinsed/removed (either as product, or as leaching) during the experiment. The magnesium content was determined by Flame Atomic Absorption Spectrophotometry, using the Perkin-Elmer AAnalyst 400 instrument. The experimental conditions, applied in the chamber, simulated the usual sewer pipe conditions, i.e., maintaining the temperature at 20 ± 2 °C and 99% relative humidity.

### 2.5. X-ray Diffraction (XRD) Analysis and Attenuated Total Reflectance (ATR)

The recognition and quantitative determination of mineralogical phases existing on the specimens’ surface were performed with the XRD analysis. After the spraying process, the specimens were dried at 40 °C for 24 h, and then the coating and the potentially formed by-products were scratched from the top of specimens, ground and measured. The structural phases (mineralogical composition) of the obtained samples were analyzed by XRD measurements, using a PW 1840 Phillips diffractometer with CuKa radiation, step size of 0.02° and step time of 0.4 s, operating at 30 kV and 10 mA. The obtained diffractograms were further quantified by following the Rietveld methodology, using the FullProf Suite Software.

ATR was additionally used for the characterization of selected surface samples from the longer duration test. For the ATR measurements a Cary 630 FTIR, Agilent Technologies and the respective software MicroLab were used. The samples were measured directly after the XRD measurements without any prior treatment.

## 3. Results

### 3.1. Adhesion Measurements

The results of pull-off measurements can offer important information about the adhesion ability of protective coatings onto an appropriate substrate. In particular, the coating’s tensile bond strength can be calculated by using the failure load, resulting from the respective measurements. Additionally, the type of failure can be determined optically, according to the aforementioned standards [33,34] and expressed as a percentage, based on the relevant surface area. Two concrete specimens, with 4 testing areas on each one, were used for the evaluation of each sample coating.

According to the standards, and regarding the pull-off measurements, several types of coating failures can be optically observed and defined, depending on the applied coatings. However, in this study, two specific types of failure were present, i.e., the adhesion failure between the substrate/concrete and the coating (denoted hereafter as A/B), and the cohesion failure within the layer of coating itself (denoted hereafter as B). The results, regarding these failure types, are usually presented as a combination between the two aforementioned types (and as percentage).

The tensile bond strength between the applied coating and the concrete substrate, as well as the type of failure, is presented in Table 3. The results of studied coating are compared with the results of other relevant coatings, previously examined [28], in order to draw useful conclusions and are commented below.

As far as the tensile bond is concerned, the coating was found to present medium-to-high values when compared to other relevant coatings, consisting of different magnesium hydroxide powders [28]. The specific type of failure plays a key role in understanding the adhesion ability of coating, when combined with the tensile bond strength. The application of examined coating generally requires high values of the B failure type, so that in case of coating detachment, a significant amount of coating can continue to protect the substrate. Therefore, the calculated values, regarding the two types of failure, are considered as satisfactory, because the coating exhibited more type-B failure (70%), than type-A/B (30%). As a result, this coating combined the required type of failure with a satisfactory tensile bond strength value, as compared with other relevant magnesium hydroxide coatings [28].

### 3.2. SEM Analysis

**Figure 1:**
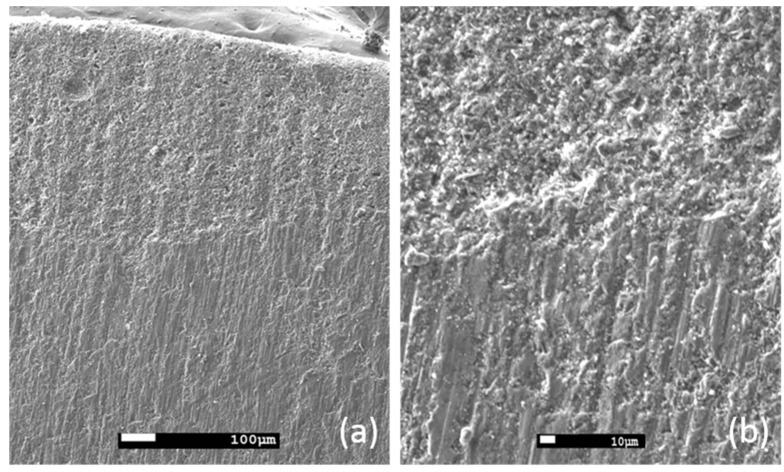
SEM micrograph of the coated concrete specimen at the interface region: magnification (**a**) ×60, (**b**) ×200.

Figure 1 is a cross-section micrograph of the coated concrete specimen, where the coating is presented in the upper side, whereas the concrete substrate is presented in the lower side of the image. The adhesion ability of the coating can be verified by this figure, because the coating seems to be firmly attached onto the concrete surface, without any voids or cracks observed at the interface area. The concrete surface is distinct, because it presents different roughness and porosity from the coating. The structure of concrete is denser than the respective of coating, which seems to be rather porous. It is important to note that the selected concrete type is of relatively low porosity. Additionally, the scratch polishing lines are rougher on the concrete’s surface, than on the coating’s surface. The coating thickness ranged between 0.4 and 0.7 mm, according to these observations. A closer look at the coating-concrete interface (Figure 1, right) confirms the good adhesion of the coating onto the surface.

**Figure 2:**
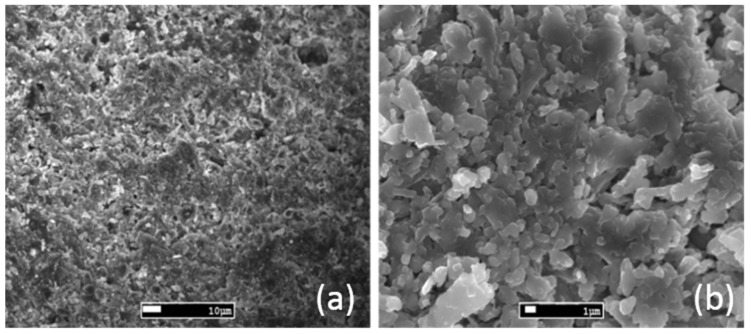
SEM micrographs regarding the structure of examined coating: magnification (**a**) ×500, (**b**) ×3000.

As far as the coating’s microstructure is concerned (Figure 2), the particles (magnesium hydroxide particles, or magnesium oxide) are mainly spherical and elongated, usually presenting a size around 1 μm. The structure is porous and loose, while the EDS analysis showed mainly the presence of magnesium (apart from oxygen).

**Figure 3:**
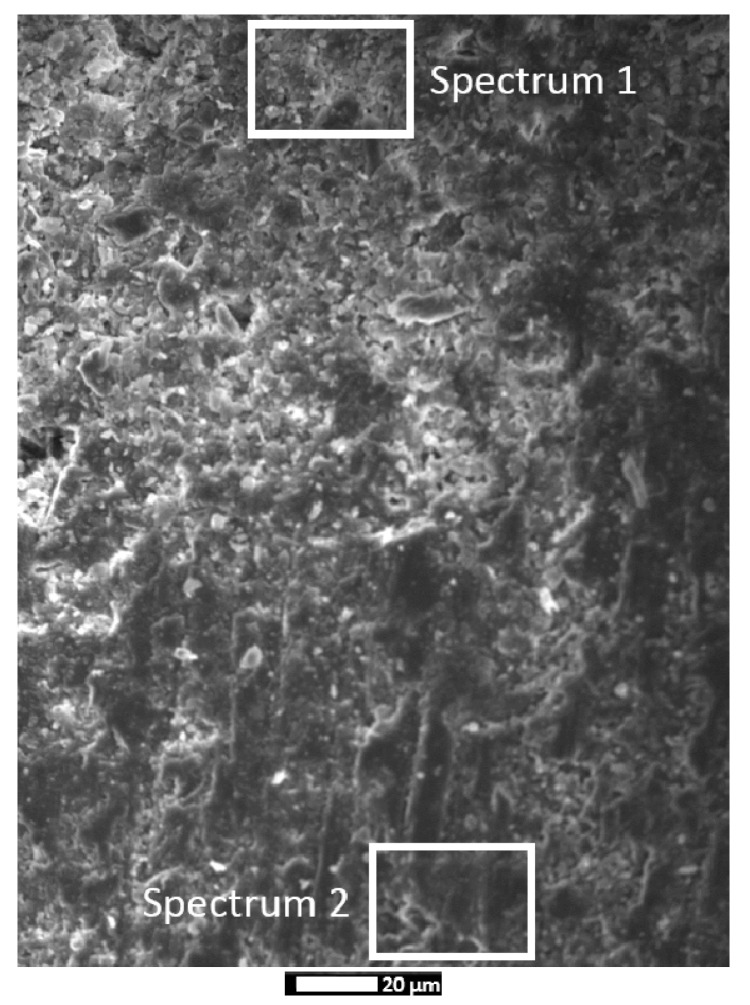
SEM micrograph of the coating-concrete interface, showing the respective energy-dispersive X-ray spectroscopy (EDS) spectral areas.

The EDS analysis of the coating and of the concrete surface can provide information regarding the potential penetration of coating constituents into the concrete structure, which can also explain the coating’s good adhesion and create an additional protection zone in the concrete structure. The EDS analysis of the spectrum areas (Figure 3) is presented in Table 4. The results showed that the magnesium content in the concrete’s structure (and in the depth of ~100 μm) is relatively small (1.41% wt.). According to the relevant literature [28], this corresponds to the initial magnesium content of concrete. In contrast to the previous study, this coating does not seem to penetrate the concrete substrate. The increased calcium content of concrete surface (Spectrum 2) is attributed to the calcium silicate compounds of the hydrated cement paste, as well as to the limestone (calcium carbonate) source of aggregates.

### 3.3. Accelerated Sulfuric Acid Spraying Tests

#### 3.3.1. 4-Days Accelerated Acid Spraying Tests

The study of coating surface pH values during its interaction with sulfuric acid is very important, because this is the main acquired and inherent property of magnesium hydroxide coating, against the biogenic corrosion of concrete. The maintenance of alkaline surface pH values can block the development of sulfur oxidizing bacteria, hence the production of biogenic sulfuric acid and the subsequent corrosion of concrete.

**Figure 4:**
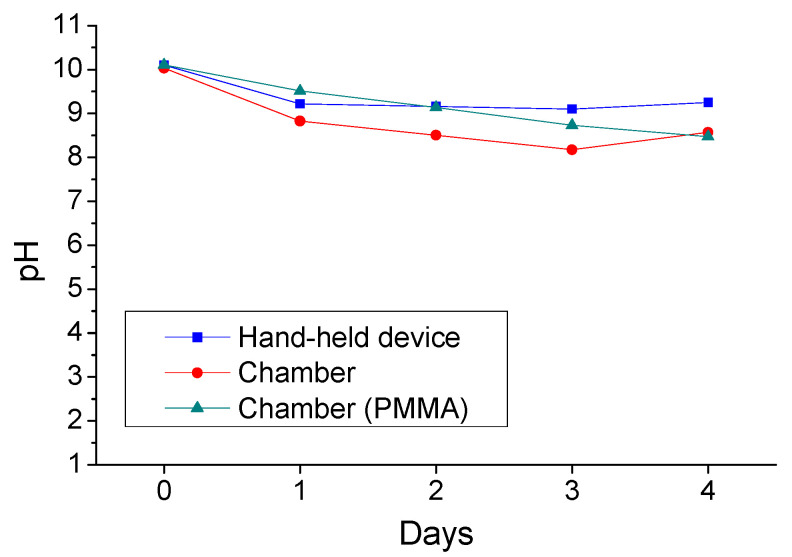
Surface pH values of coated concrete and PMMA specimens, during the application of different acid spraying tests.

The daily recordings of surface pH values, regarding the coated concrete specimens, when sprayed during the HHD test, as well as the coated concrete specimens sprayed in the spraying chamber are presented in Figure 4. Moreover, the surface pH values of the coated PMMA plates are also presented in Figure 4.

In all these cases, the starting pH values of the coating, measured on the substrates’ surface, were very similar. The coating presented initial surface pH values around value 10 (day 0). Despite the differences between the tests, the application of acid spraying led to slightly decreased surface pH values (i.e., 8.00–9.25), comparing with the initial pH values. However, the pH values remained in the alkaline region throughout the spraying processes, which means that the coating can block effectively the development of bacteria. The differences of pH values between the tests found to be relatively small. More precisely, after four days of spraying, the values measured for the specimens, sprayed under the HHD test, were the highest, i.e., 9.25. This fact may be due to the smaller number of spraying applications in the case of HHD test, even though a highly concentrated sulfuric acid solution was used, as compared with the number of spraying applications in the case of the chamber test.

The respective pH values of coated PMMA plates were found to be slightly higher, at days 1, 2, and 3 of chamber spraying test, with respect to the values of coated concrete specimens. The comparison of surface pH values for the coating, during the chamber test, as applied either on concrete substrates (dry coating), or on PMMA plates, indicates that the presence of concrete substrate might also influence the measured pH values.

These values can be compared with the corresponding ones of other magnesium hydroxide coatings, as well as of uncoated concrete specimens, which were sprayed accordingly [28]. The examined coating exhibited satisfying surface pH values (pH > 8) in most cases of spraying tests, although the initial pH value of it at day 0 was not the highest among the several relevant coatings.

**Figure 5:**
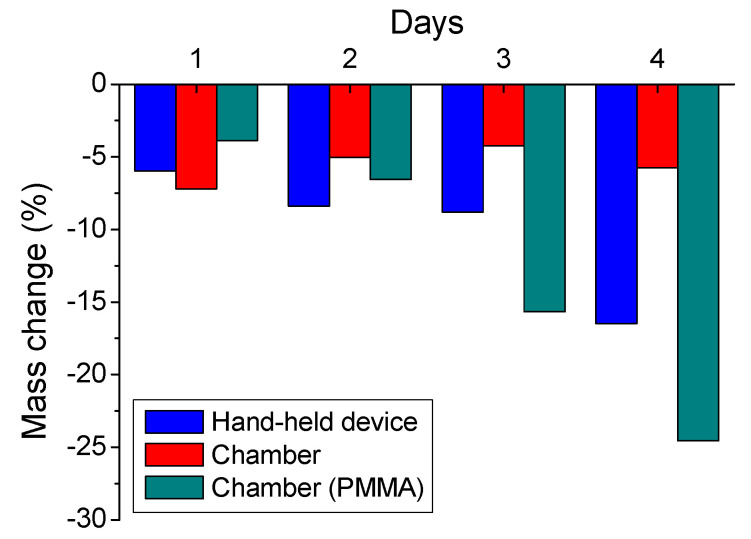
Mass changes of the coated concrete specimens after the application of HHD spraying test, as well as of the coated concrete and PMMA plates after the application of chamber spraying test.

Moving on, mass measurements were also conducted for the coated concrete specimens subjected to both tests (HHD and chamber), as well as for the coated PMMA plates (chamber test). The results are presented as percentage, in respect to the initial coating mass and are given in Figure 5.

According to the stoichiometry calculations, a larger consumption of the coating, at all these cases, can be expected, because the amount of sulfuric acid that was sprayed onto the specimens, during the 4-days tests, corresponded to the coating’s total magnesium hydroxide amount. However, it can be assumed that the sprayed sulfuric acid can only react with the exposed coating surface, instead of the full coating’s mass [28], somehow reducing the mass consumption respectively.

The mass change results indicated that the substrate affects the behavior of the coating concerning mass change. The mass change of the coating on the concrete specimens (chamber test) presented rather stable behavior during the spraying procedure. On the other hand, the mass change of coating on the PMMA specimens was more intensified during the test. In this case the mass decrease reached almost 25%, when compared to the initial mass of the system. It seems possible that the adhesion between the coating and the concrete can play an important role on the system’s behavior. In this respect the porosity of concrete and of concrete surface are important parameters. On the contrary, the absence of adhesion between the used coating and the polymer substrate (PMMA) did not contribute to that direction.

The concentration of sprayed sulfuric acid can also play an important role, as during the HHD test (using the higher concentration of 4 M), the mass loss was greater than in the chamber test (using the lower sulfuric acid concentration of 0.1 M). This reasonable result is connected to the greater consumption of coating in the case of greater acid concentration sprayed.

It is also a fact that mass increase was not observed, as it was recorded for some other magnesium hydroxide coatings, presenting similar characteristics [28]. This is connected to the washing out rate of reaction (neutralization) by-products and the ease that the particular coating behaves under the applied spraying conditions, as its behavior depends mainly on its specific physicochemical characteristics, such as the smaller particle size and the higher surface area in comparison with other relevant materials, previously examined [28].

**Figure 6:**
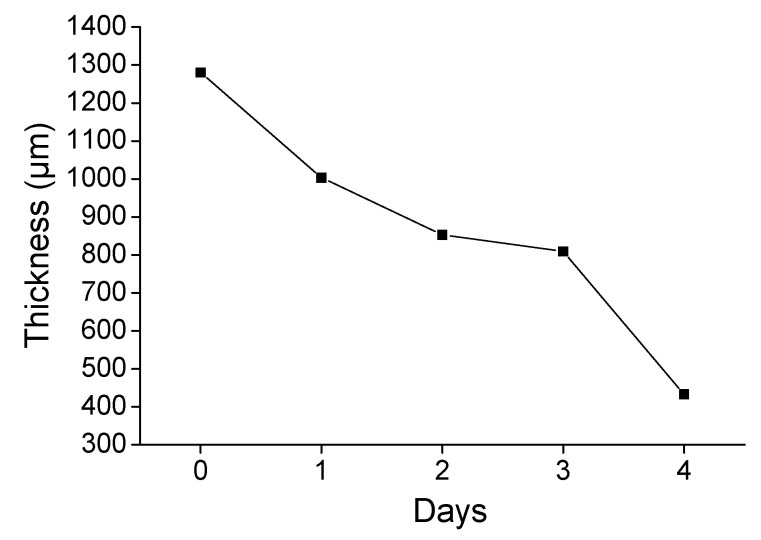
The evolution of thickness coating throughout the HHD spraying test.

The measurement of coating thickness during the HHD acid spraying test was performed as another way to evaluate the coating consumption under the application of extreme acidic conditions (high concentration of sprayed sulfuric acid). The coating thickness was expected to decrease during the spraying procedure, due to its reaction with sulfuric acid. The results presented in Figure 6 confirmed that the coating thickness is gradually reduced, due to the coating’s consumption. In this case the thickness reduction of studied coating was higher (−66%), than the thickness reduction of other studied relevant coatings [28]. This fact indicated that the coating was more effectively consumed, also confirming the mass change results. Additionally, the mass loss, intensified at day 4 of the experiment, agreed with the extended decrease of thickness at the same day.

#### 3.3.2. 4-Months Accelerated Acid Spraying Test

**Figure 7:**
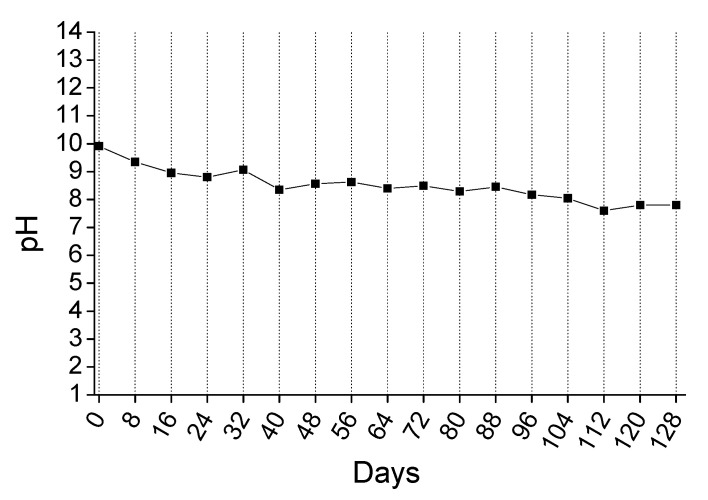
The evolution of surface pH values of coated concrete specimens, during the application of the long-term duration acid spraying test.

The 4-months accelerated acid spraying test was performed to better simulate the existing conditions in a sewer, as described in Section 2.4.3. The surface pH, the mass change, and the mineralogical phases of surface were recorded. Figure 7 presents the results of surface pH recordings of the sprayed specimens at 8-day intervals. The surface pH values were maintained at the alkaline region throughout the long duration test and confirmed the ability of the coating to block the development of acidophilic sulfur oxidizing bacteria. In that way, only the neutrophilic sulfur oxidizing bacteria might be developed and produce a small amount of biogenic sulfuric acid. Nevertheless, based on the previous results (Section 3.3.1), the coating can still react with the acid and protect the concrete surface.

The variation of pH values during the 128 days experiment were quite similar to those obtained from the 4-day testing, considering the respective acid concentrations. This shows that the short time tests are also reliable and both tests (long- or short-term) can be performed to evaluate the pH behavior of this magnesium-based coating.

**Figure 8:**
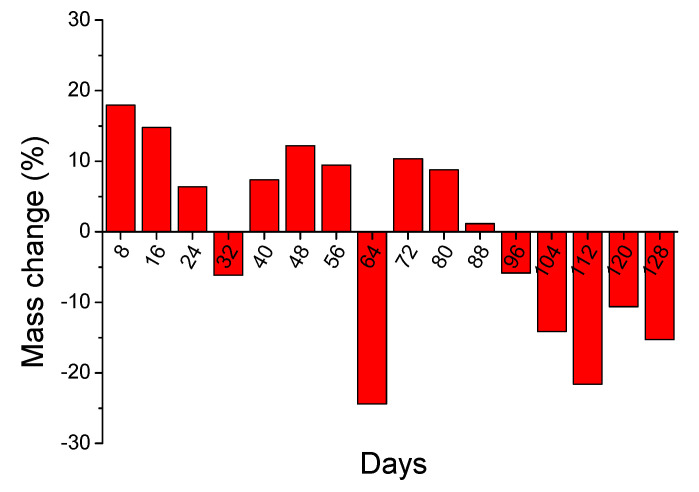
The evolution of mass changes of coated concrete specimens during the application of long-term duration acid spraying test.

Moving on, the mass change results of the long duration acid spraying test are presented in Figure 8. The results are shown in respect to the initial coating mass (%) applied on the concrete specimens. The complicated mechanisms that took place during the spraying applications, led to rather controversial mass change results. Two different processes can take place during the acid spraying test, i.e., the reaction (neutralization) of the coating with the sprayed acid and the formation of by-products (mainly hexahydrite and gypsum). The first process leads to mass decrease, while the second one to mass increase. It is also important to note that both processes can take place simultaneously.

According to the results, the mass of specimens increased (positive values) during the first 80 days, with the exceptions of specimens at day 32 and day 64. Regarding the results of day 32 specimens, there was an unregular mass decrease, with respect to the trend of the previous and the following specimen results. However, the results are given for different specimens each time, and this can lead to different results in some cases. Regarding the results of day 64, there was a technical problem during the test and mass loss was observed, due to coating detachment. After 88 days of acid spraying, mass decrease was recorded until the end of the procedure.

These results, and especially the mass increase, differed from the respective tests of the accelerated acid spraying tests (Figure 5), where no mass increase was observed. However, other magnesium hydroxide coatings presented also mass increase, during the accelerated acid spraying test, which was attributed to the formation of by-products [28]. It is a fact that the experimental conditions of the 4-months test were different, i.e., the solution concentration and the duration and the number of daily spraying applications. Accordingly, these factors enabled the long-term milder condition testing, which allowed the recording of initial increase of mass, due to the formation of by-products and the following decrease of mass, due to the consumption/degradation of coating. Based on that, both mechanisms took place. The possible formation of by-products was further examined using the XRD measurements (Section 3.4.2).

**Figure 9:**
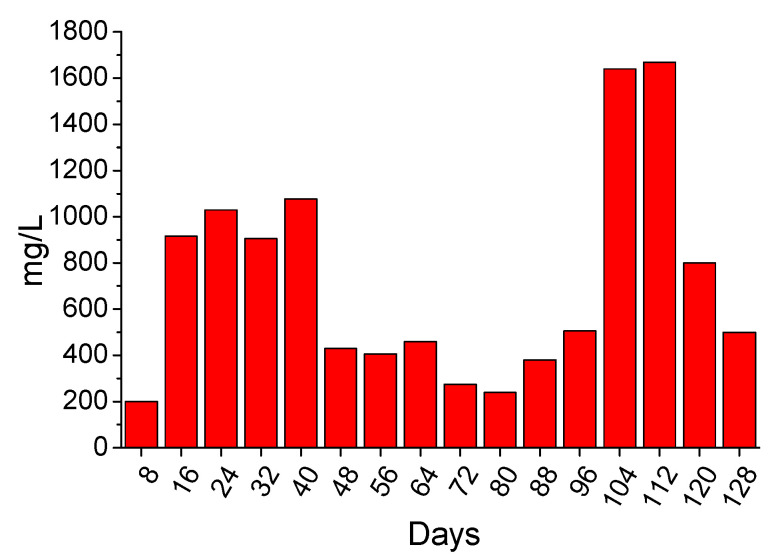
The evolution of magnesium content (mg/L) of rinsing liquids at 8-days intervals during the application of long duration acid spraying test.

The long-term acid spraying test was also conducted in a poly (methyl methacrylate) chamber, which was modified in a way that the washouts could be collected and analyzed. After that, the magnesium content was determined in them, and the results are presented in Figure 9. The presence of magnesium in the rinsing liquids indicated the continuous reaction of magnesium hydroxide with the sprayed acid or its possible leaching. The magnesium content results seem to agree with the mass change results, because, in general, when mass decrease was observed (e.g., at day 112), there was an increase of magnesium content in the rinsing liquids. However, magnesium was detected, even when the mass increase was recorded. This indicates that an amount of magnesium reacted and was released in the liquid phase even at the initial sprayed days.

According to the above-mentioned results, the long duration test showed that the examined coating was consumed during the experimental procedure. The by-products can be evaluated by the following XRD results.

### 3.4. XRD Analysis

#### 3.4.1. 4-Days Accelerated Acid Spraying Tests

**Figure 10:**
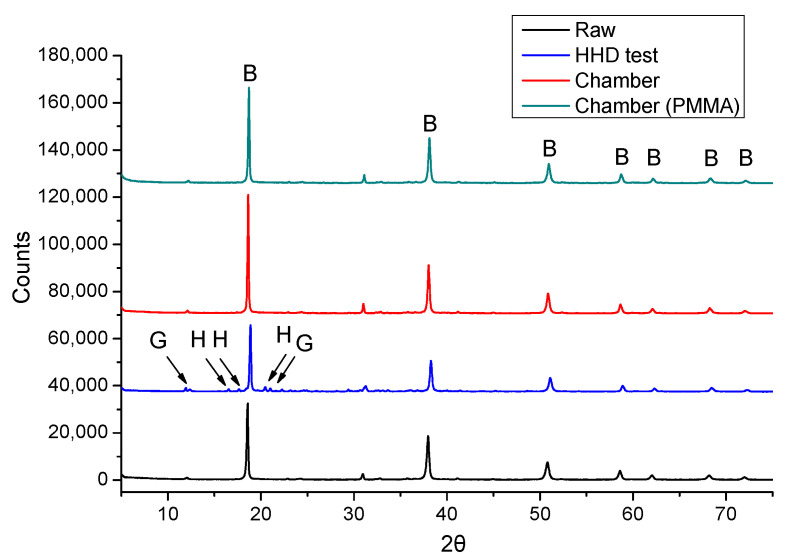
XRD overlay diffractograms of coated specimens after 4 days of different acid spraying tests’ application (the raw material is also presented); B: Brucite, G: Gypsum, H: Hexahydrite.

The respective samples were examined by using XRD and the respective results are presented as XRD diffractograms overlay. Figure 10 shows the results regarding the coated specimens after 4 days of each acid spraying test, i.e., the HHD test, the chamber test using coated concrete specimens and the chamber test using PMMA plates as substrate. Moreover, the diffractogram of the raw material, i.e., without being subjected to acid spraying, is also presented in the same Figure for comparison reasons.

**Figure 11:**
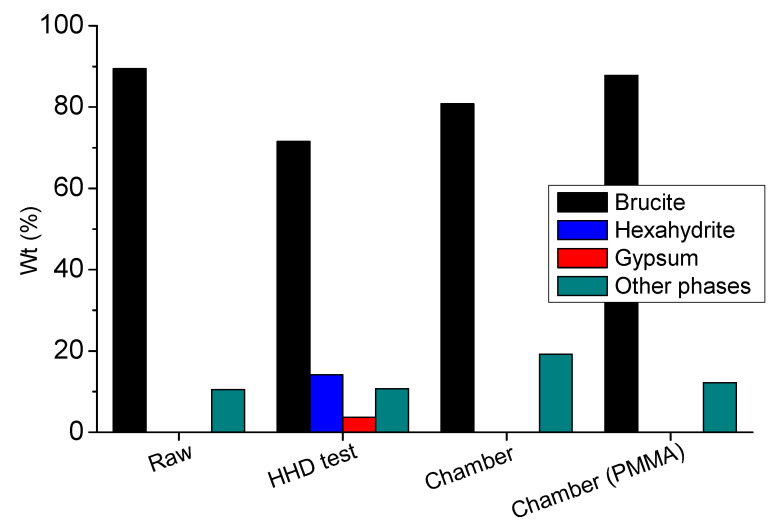
The semi-quantitative results of XRD analysis, regarding the presence of crystalline phases for the coated specimens after 4 days of each acid spraying test applications; «raw» is the coating but without being subjected to spraying applications.

According to these results, the main peaks of brucite (Mg(OH)_2_) are indicated, whereas the peaks of other phases (i.e., lizardite (Mg_3_(Si_2_O_5_)(OH)_4_) and dolomite (Ca,Mg(CO_3_)_2_)) are not easily indicated (presenting low intensity), but they are also included in the semi-quantitative results presented in Figure 11. The main corrosion by-products, i.e., gypsum (CaSO_4_·2H_2_O) and hexahydrite (MgSO_4_(H_2_O)_6_), are indicated in Figure 10, but only for the case of HHD test. This fact could be attributed to the different frequency of applied spraying and the higher acid concentration, as these were the main differences between the applied tests. The concentration of sulfuric acid during the HHD test was higher (4 M), hence it may lead to the immediate formation of by-products, as comparing to the other tests’ samples, where much lower sulfuric acid concentration was used; noting also that the real conditions, existing in sewer pipe (“crown”) surface, are much milder, than the high concentrations used during the HHD test. The lower applied concentration of acid solution during the chamber tests, allowed the faster reaction of the coating with the acid; hence preventing the diffusion of acid through the coating. As a result, the concrete surface was not affected, and no corrosion products were formed in the later cases, i.e., during the chamber tests.

It is important to note that the formed magnesium sulfate (hexahydrite) is water soluble and can be easily washed out, due to spraying applications; hence, it cannot always be traced. The differences between the tested specimens, when compared to the raw (initial) material used, indicate the formation of by-products, specifically in the case of HHD test sample. Additionally, the substrate did not seem to interfere with the found crystallographic phases results, as no peaks of concrete phases were detected, such as calcite or quartz.

The semi-quantified results of XRD diffractograms (Figure 11) confirm the presence of main by-products (gypsum and hexahydrite) in the HHD test samples. It can be also observed that brucite was in excess quantities in all accelerated tests, indicating that the coating could offer protection to the substrate, even after the end of 4-day spraying test procedures.

Due to the two different sources of calcium, i.e., from the concrete or from the coating, it is not clear whether gypsum was a corrosion by-product of the substrate (concrete), or of the coating content only. In that way, the tests using PMMA plates were also performed to verify the origin of potential gypsum formation. Regarding the respective results of PMMA plates, gypsum formation was not observed, concluding that the formed gypsum during the HHD tests corresponded to the corrosion of concrete. In comparison, the results of the chamber tests indicate that the coating can provide anti-corrosion protection to the concrete substrate, because gypsum formation was better prevented in this case.

#### 3.4.2. 4-Months Accelerated Acid Spraying Test

**Figure 12:**
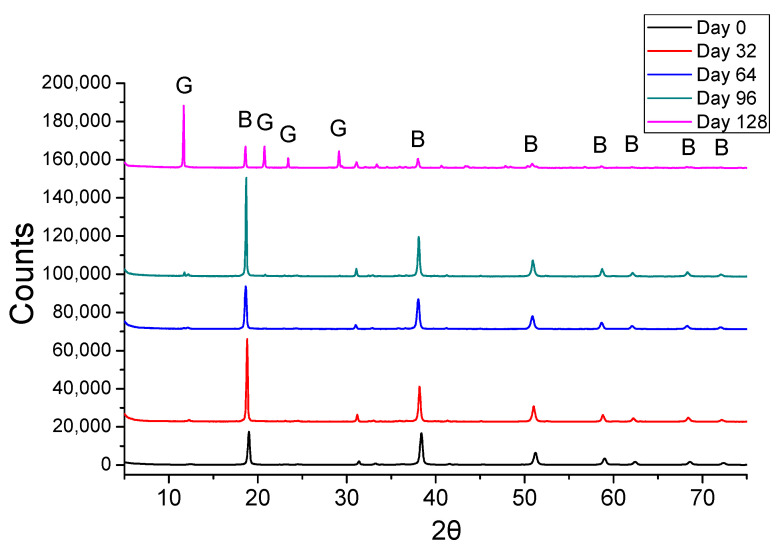
XRD overlay diffractograms of selected coated concrete specimens during the application of the 4-months acid spraying test; G: Gypsum, B: Brucite.

**Figure 13:**
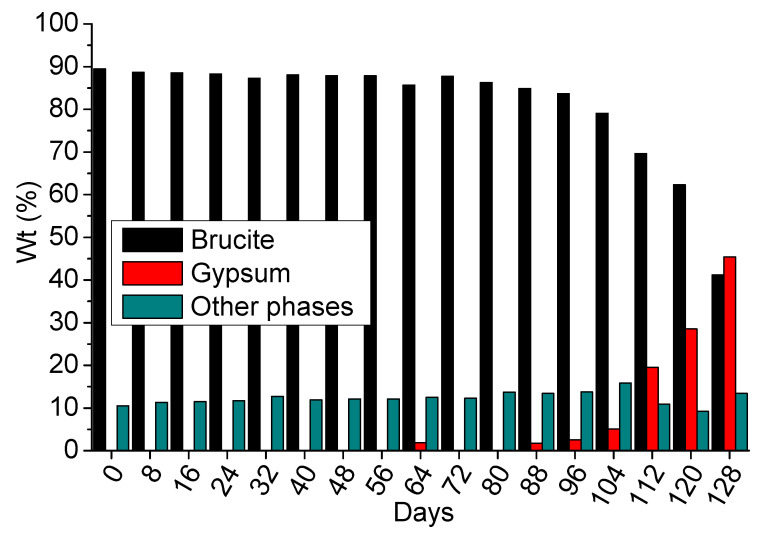
The semi-quantitative results of XRD analysis, regarding the crystalline phases of coated concrete specimens during the application of 4-months acid spraying test.

The coating was examined under sulfuric acid spraying in a spraying chamber test for 4 months, using 0.1 M sulfuric acid solution. The selected XRD diffractograms at days 0, 32, 64, 96, and 128 are presented in Figure 12. The peaks of brucite were identified, along with the gypsum for the samples at days 96 and 128. No hexahydrite peaks were identified in any sample. Additionally, the peaks of the other phases are rather small to be indicated, but they were included in the respective semi-quantitative results presented in Figure 13.

The semi-quantitative XRD results of all eight-day intervals are presented in Figure 13. The crystalline phase of brucite (coating) was found in excess until the sample of day 120. This result indicates that the coating can protect the substrate almost for the time duration that it was theoretically calculated; noting that the total amount of sprayed acid was calculated in order to consume the whole coating mass. Therefore, it can be expected that gypsum would be formed, due to the consumption of protective coating and the subsequent reaction with the exposed concrete surface before the end of this test.

The gypsum crystalline phase was identified at the sample of day 64, but at low content (i.e., close to experimental error), although the periodic increase of gypsum was recorded even after the 88th day. The gypsum at day 64 might be formed due to sample weaknesses and structure deformation, creating defects and leading to the detachment of coating.

According to these results, the coating can practically offer long-term anti-corrosion protection to the concrete surface. The coating protected the concrete surface for at least the 87.5% of the calculated theoretical time without allowing the formation of significant gypsum by-product. After day 112, the degradation of concrete is recorded, due to gypsum formation, despite the maintained alkaline pH values.

### 3.5. Attenuated Total Reflectance

**Figure 14:**
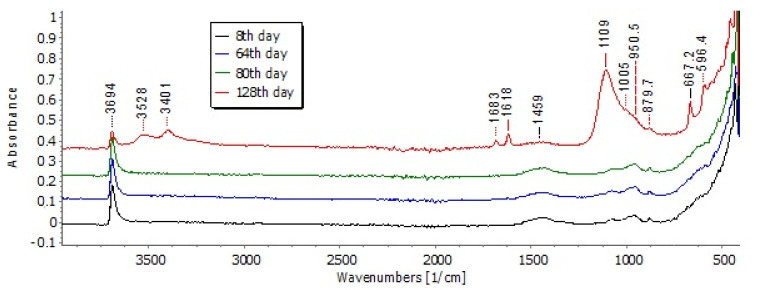
The evolution of ATR spectra of selected samples at 8, 64, 80 and 128 days during the long-term spraying test.

The ATR measurements record the vibrations of bonds of the compounds and the respective spectra are displayed in Figure 14. The representative spectra were selected to reveal the progress of gypsum formation. In particular, the spectra of samples at 8, 64, 80, and 128 days are presented. The stretching of hydroxylic groups of magnesium hydroxide bonds (brucite) is shown at the wavenumber 3694 cm^−1^ [35]. The peaks, due to carbonate ion, appear at 1459 cm^−1^ and 879 cm^−1^, which implies that carbonated species are also present in the samples, although the relevant IR response is rather weak.

At the 128th day spectrum gypsum peaks were identified. The strong peak at 1109 cm^−1^ is attributed to sulphates (SO_4_^−2^) [36]. Additionally, the band at 1683 cm^−1^ and 1618 cm^−1^ is attributed to the O-H bending vibrations of water molecules [35,37]. The sharp peaks at 667 cm^−1^ and 596.4 cm^−1^ are owed to the stretching and bending modes of sulfate group of the gypsum [37]. Gypsum is formed and recorded at later dates of the long-term testing, showing that the ATR measurements are in good agreement with the aforementioned XRD results.

## 4. Discussion

According to the previous experimental study [28], the characteristics of magnesium hydroxide-based coatings for the protection of concrete surfaces can determine its behavior and effectiveness. The particle size, purity, and specific surface area of raw material (i.e., the initial magnesium oxide powder) and the additives used for the slurry preparation can influence the performance of the coating. The studied magnesium hydroxide was designed to produce a coating that can offer better anti-corrosion protection in comparison with other relevant materials, when applied on the (internal) surface of concrete sewer pipes. The PSD and the purity of used hydroxide were selected, according to the results of the relevant previous study, while the SSA was selected to be low, in order to achieve the desired (low) viscosity of the slurry, as this parameter did not seem to affect particularly the resulting anti-corrosion properties of coating [28].

The particle size of magnesium hydroxide was selected to be relatively small in order to enforce the interacting ability of resulting coating, based on the results of the previous relevant research [28]. Although the studied hydroxide presents a smaller particle size than the previous samples, the presence of hexahydrite was found to be in a smaller amount than expected, due to washing out. Hand-held testing resulted in hexahydrite and gypsum formation on the surface of coated specimens as a result of higher acid concentration that was directly sprayed on this surface (Figure 11).

Another crucial property that was selected according to previous evaluation [28] was the purity of used raw material. The magnesium hydroxide content in this case was higher, and it added to the better preservation of alkaline surface values. According to pH measurements, the values were maintained above pH 8.0 in all examined cases.

The slurry was applied on concrete specimens for long- and short-time evaluation of its resistance during acid spraying, as well as on PMMA specimens for the study of coating reaction (neutralization with sulfuric acid), but without the interference of concrete substrate.

The resulting surface pH of studied coating was maintained in the alkaline region (i.e., pH > 8.0), regardless the duration of testing and the concentration of sprayed acid. Furthermore, the coating maintained the pH to the alkaline region, when tested on both PMMA and concrete substrates and for long period of time (128 days), by applying lower acid concentration, as well as for shorter period of time (4 days), by applying higher acid concentration. The latter indicates the resistance of coating to neutralization for the aforementioned different experimental conditions.

The adhesion between the coating and the concrete surface seems to result in a system, where the two materials can “co-operate” well and the coating protects efficiently and effectively the concrete substrate. The respective mass change results pictured a gradual removing of coated material and confirmed that most probably hexahydrite was washed out, as the mass of coatings was decreased in both cases, i.e., for the concrete and PMMA substrates.

The long-term testing revealed the behavior of coating under approximately real conditions, existing in sewer pipes. The simulation of lower acid concentration (0.1 M) showed that efficient protection can be achieved. The gypsum formation was prevented, according to the XRD and ATR results. More specifically, gypsum was identified by XRD at the 112th day of spraying and ATR verified its late formation. Therefore, the coating can protect completely the concrete for this time period, under the applied experimental conditions. After 112 days the gypsum formation started, accompanied with extended brucite consumption, which indicates that the coating’s degradation leads to gypsum formation, i.e., strong evidence that the concrete is no longer protected. The magnesium removal from the surface and its leaching in washouts seemed to follow a nonlinear behavior, as it was found quite intensive during the initial experimental procedure (i.e., 10–40 days) and later (i.e., after 104 days), according to the quantification of Mg content.

The comparison of different substrates coated with the examined material revealed the gypsum source. The XRD results of PMMA coated plates did not show any gypsum formation. As a result, the gypsum formation does not originate from the calcium content of coating material, but it is attributed to the corrosion of concrete surface, due to the presence (spraying) of sulfuric acid. Additionally, it was proven that the coating’s adhesion onto the concrete’s surface also plays an important role on the consumption of coating.

## 5. Conclusions

The magnesium hydroxide slurry was found to protect the concrete from MIC by effectively maintaining alkaline surface pH values and reacting with (neutralizing) the sprayed sulfuric acid. The results of the present study indicated that the medium particle size of the raw magnesium hydroxide material used is optimal for the studied experimental conditions, in order to achieve adequate interacting ability with the sprayed sulfuric acid. Improved purity can also enhance the alkaline and anti-corrosion properties of produced coating. Moreover, the SSA property seemed to be rather independent of the anti-corrosion behavior of coating.

Corrosion by-product (i.e., gypsum) was only formed after spraying with higher concentration of sulfuric acid, during the hand-held device accelerated acid spraying test (i.e., 4 M). However, during the accelerated tests using lower sulfuric acid concentration (i.e., closer to the conditions existing in a real sewer pipe), no production of gypsum was observed. The long duration (accelerated) acid spraying test indicated that the respective coating can protect the concrete substrate, by reacting (consuming) with sulfuric acid, under conditions that can simulate the sewer pipe environment.

To sum up, the improved properties of magnesium hydroxide raw material can enhance the anti-corrosion behavior of produced coating and preserve effectively the properties of concrete substrate, even during long-term test procedures, and as a result also when used in actual sewer pipes.

## Figures and Tables

**Table 1:** The main physicochemical characteristics of used slurry solids.

Nominal Chemical Composition					Mg(OH)_2_ Content (%)	SSA (m^2^/g)	PSD (μm)	
MgO (%)	SiO_2_ (%)	CaO (%)	Fe_2_O_3_ (%)	LOI (%)			d_50_	d_90_
62.81	4.25	2.46	0.25	30.11	89.0	7	3.8	13.1

**Table 2:** The acid spraying tests and the respective measurements.

Test	Measurements
4 days hand-held device test	Surface pH Thickness Mass Mineralogical phases
4 days spraying chamber test (concrete specimens)	Surface pH Mass Mineralogical phases
4 days spraying chamber test (PMMA plates)	Surface pH Mass Mineralogical phases
128 days spraying chamber test	Surface pH Mass Mineralogical phases

**Table 3:** Tensile bond strength (f_h_) values, the respective standard deviation (SD) and the specific type of coatings’ failure.

f_h_ (MPa)	SD (MPa)	Type of Failure	
		A/B (%) ^1^	B (%) ^2^
0.30	0.025	30	70

^1^ Adhesion failure between the substrate and the coating layer; ^2^ Cohesion failure within the layer of coating.

**Table 4:** EDS spectra of the coating and of the concrete, as presented in Figure 3.

Elements	Weight %	
	Spectrum 1	Spectrum 2
Mg	94.16	1.41
Si	2.31	1.24
Ca	3.54	97.35

## Data Availability

The data underlying this article will be shared on reasonable request from the corresponding author.
